# Supplementary material for: Examination of a first-in-class bis-dialkylnorspermidine-terphenyl antibiotic in topical formulation against mono and polymicrobial biofilms
Source: PLoS One. 2020 Oct 19;15(10):e0234832. doi: 10.1371/journal.pone.0234832 (PMC7571676; doi:10.1371/journal.pone.0234832)
Supplement: S1 Table — (PDF) [file pone.0234832.s001.pdf]

Supplemental Table 1: Raw microbiological data for MRSA

Treatment	CFU	Dilution Factor 1 (scientific notation)	Dilution Factor 2 (1:20 for final pipet step)	CFU/mL	CFU/sample	Log_{10} Transformed	Average	Standard Deviation
Collagen (+ controls)	22	1.00E+06	0.03	7.33E+08	1.47E+09	9.16633142	9.37316817	0.301686579
	48	1.00E+06	0.03	1.60E+09	3.20E+09	9.50514998		
	16	1.00E+06	0.03	5.33E+08	1.07E+09	9.02802872		
	15	1.00E+06	0.03	5.00E+08	1.00E+09	9.00000000		
	22	1.00E+06	0.03	7.33E+08	1.47E+09	9.16633142		
	43	1.00E+06	0.03	1.43E+09	2.87E+09	9.45737720		
	21	1.00E+06	0.03	7.00E+08	1.40E+09	9.14612804		
	93	1.00E+06	0.03	3.10E+09	6.20E+09	9.79239169		
	104	1.00E+06	0.03	3.47E+09	6.93E+09	9.84094208		
	112	1.00E+06	0.03	3.73E+09	7.47E+09	9.87312676		
	67	1.00E+06	0.03	2.23E+09	4.47E+09	9.64998354		
	36	1.00E+06	0.03	1.20E+09	2.40E+09	9.38021124		
	40	1.00E+06	0.03	1.33E+09	2.67E+09	9.42596873		
	85	1.00E+06	0.03	2.83E+09	5.67E+09	9.75332767		
	16	1.00E+06	0.03	5.33E+08	1.07E+09	9.02802872		
	23	1.00E+06	0.03	7.67E+08	1.53E+09	9.18563658		
	18	1.00E+06	0.03	6.00E+08	1.20E+09	9.07918125		
	26	1.00E+06	0.03	8.67E+08	1.73E+09	9.23888209		
Silver Sulfadizine 1%	22	1.00E+03	0.03	7.33E+05	1.47E+06	6.16633142	6.40413276	0.341016982
	50	1.00E+03	0.03	1.67E+06	3.33E+06	6.52287875		
	31	1.00E+03	0.03	1.03E+06	2.07E+06	6.31527043		
	53	1.00E+03	0.03	1.77E+06	3.53E+06	6.54818461		
	106	1.00E+03	0.03	3.53E+06	7.07E+06	6.84921461		
	44	1.00E+03	0.03	1.47E+06	2.93E+06	6.46736142		
	8	1.00E+03	0.03	2.67E+05	5.33E+05	5.72699873		
	65	1.00E+03	0.03	2.17E+06	4.33E+06	6.63682210		
Neosporin	31	1.00E+04	0.03	1.03E+07	2.07E+07	7.31527043	6.91057647	0.524516559
	36	1.00E+03	0.03	1.20E+06	2.40E+06	6.38021124		
	26	1.00E+03	0.03	8.67E+05	1.73E+06	6.23888209		
	18	1.00E+04	0.03	6.00E+06	1.20E+07	7.07918125		
	35	1.00E+04	0.03	1.17E+07	2.33E+07	7.36797679		
	27	1.00E+03	0.03	9.00E+05	1.80E+06	6.25527251		
	40	1.00E+04	0.03	1.33E+07	2.67E+07	7.42596873		
	25	1.00E+04	0.03	8.33E+06	1.67E+07	7.22184875		
Gentamicin 1%	30	1.00E+02	0.05	6.00E+04	1.20E+05	5.07918125	5.81102919	0.561470293
	30	1.00E+02	0.05	6.00E+04	1.20E+05	5.07918125		
	57	1.00E+02	0.05	1.14E+05	2.28E+05	5.35793485		
	29	1.00E+03	0.05	5.80E+05	1.16E+06	6.06445799		
	64	1.00E+03	0.05	1.28E+06	2.56E+06	6.40823997		
	19	1.00E+03	0.05	3.80E+05	7.60E+05	5.88081359		
	44	1.00E+03	0.05	8.80E+05	1.76E+06	6.24551267		
	59	1.00E+03	0.05	1.18E+06	2.36E+06	6.37291200		
Mupirocin 2%	51	1.00E+03	0.03	1.70E+06	3.40E+06	6.53147892	6.20533873	0.521414399
	98	1.00E+03	0.03	3.27E+06	6.53E+06	6.81513482		
	16	1.00E+03	0.03	5.33E+05	1.07E+06	6.02802872		
	52	1.00E+03	0.03	1.73E+06	3.47E+06	6.53991208		
	47	1.00E+03	0.03	1.57E+06	3.13E+06	6.49600660		
	32	1.00E+03	0.03	1.07E+06	2.13E+06	6.32905872		

	6	1.00E+03	0.03	2.00E+05	4.00E+05	5.60205999		
	3	1.00E+03	0.03	1.00E+05	2.00E+05	5.30103000		
**Retapamulin 1%**	73	1.00E+04	0.05	1.46E+07	2.92E+07	7.46538285	7.74872351	0.354209982
	56	1.00E+04	0.05	1.12E+07	2.24E+07	7.35024802		
	100	1.00E+04	0.05	2.00E+07	4.00E+07	7.60205999		
	47	1.00E+05	0.05	9.40E+07	1.88E+08	8.27415785		
	34	1.00E+05	0.05	6.80E+07	1.36E+08	8.13353891		
	13	1.00E+05	0.05	2.60E+07	5.20E+07	7.71600334		
	65	1.00E+04	0.05	1.30E+07	2.60E+07	7.41497335		
	27	1.00E+05	0.05	5.40E+07	1.08E+08	8.03342376		
**CZ-01179 0.5%**	0	1.00E+00	0.03	0.00E+00	0.00E+00	0	0.00000000	0
	0	1.00E+00	0.03	0.00E+00	0.00E+00	0		
	0	1.00E+00	0.03	0.00E+00	0.00E+00	0		
	0	1.00E+00	0.03	0.00E+00	0.00E+00	0		
	0	1.00E+00	0.03	0.00E+00	0.00E+00	0		
	0	1.00E+00	0.03	0.00E+00	0.00E+00	0		
	0	1.00E+00	0.03	0.00E+00	0.00E+00	0		
	0	1.00E+00	0.03	0.00E+00	0.00E+00	0		
**CZ-01179 1%**	0	1.00E+00	0.03	0.00E+00	0.00E+00	0	0.00000000	0
	0	1.00E+00	0.03	0.00E+00	0.00E+00	0		
	0	1.00E+00	0.03	0.00E+00	0.00E+00	0		
	0	1.00E+00	0.03	0.00E+00	0.00E+00	0		
	0	1.00E+00	0.03	0.00E+00	0.00E+00	0		
	0	1.00E+00	0.03	0.00E+00	0.00E+00	0		
	0	1.00E+00	0.03	0.00E+00	0.00E+00	0		
	0	1.00E+00	0.03	0.00E+00	0.00E+00	0		
**CZ-01179 2%**	0	1.00E+00	0.03	0.00E+00	0.00E+00	0	0.00000000	0
	0	1.00E+00	0.03	0.00E+00	0.00E+00	0		
	0	1.00E+00	0.03	0.00E+00	0.00E+00	0		
	0	1.00E+00	0.03	0.00E+00	0.00E+00	0		
	0	1.00E+00	0.03	0.00E+00	0.00E+00	0		
	0	1.00E+00	0.03	0.00E+00	0.00E+00	0		
	0	1.00E+00	0.03	0.00E+00	0.00E+00	0		
	0	1.00E+00	0.03	0.00E+00	0.00E+00	0		
