# Supplementary material for: Examination of a first-in-class bis-dialkylnorspermidine-terphenyl antibiotic in topical formulation against mono and polymicrobial biofilms
Source: PLoS One. 2020 Oct 19;15(10):e0234832. doi: 10.1371/journal.pone.0234832 (PMC7571676; doi:10.1371/journal.pone.0234832)
Supplement: S2 Table — (PDF) [file pone.0234832.s002.pdf]

Supplemental Table 2: Raw microbiological data for *Pseudomonas aeruginosa* (ATCC 27853)

Treatment	CFU	Dilution Factor 1 (scientific notation)	Dilution Factor 2 (1:20 for final pipet step)	CFU/mL	CFU/sample	Log_{10} Transformed	Average	Standard Deviation
Collagen (+ controls)	55	1.00E+05	0.03	1.83E+08	3.67E+08	8.56427143	8.52243387	0.342332528
	46	1.00E+05	0.03	1.53E+08	3.07E+08	8.48666657		
	25	1.00E+05	0.03	8.33E+07	1.67E+08	8.22184875		
	18	1.00E+06	0.03	6.00E+08	1.20E+09	9.07918125		
	56	1.00E+05	0.03	1.87E+08	3.73E+08	8.57209677		
	46	1.00E+05	0.03	1.53E+08	3.07E+08	8.48666657		
	39	1.00E+05	0.03	1.30E+08	2.60E+08	8.41497335		
	65	1.00E+05	0.03	2.17E+08	4.33E+08	8.63682210		
	25	1.00E+05	0.03	8.33E+07	1.67E+08	8.22184875		
	52	1.00E+05	0.03	1.73E+08	3.47E+08	8.53991208		
	44	1.00E+06	0.03	1.47E+09	2.93E+09	9.46736142		
	15	1.00E+05	0.03	5.00E+07	1.00E+08	8.00000000		
	41	1.00E+05	0.03	1.37E+08	2.73E+08	8.43669260		
	23	1.00E+05	0.03	7.67E+07	1.53E+08	8.18563658		
	47	1.00E+05	0.03	1.57E+08	3.13E+08	8.49600660		
	34	1.00E+05	0.03	1.13E+08	2.27E+08	8.35538766		
	78	1.00E+05	0.03	2.60E+08	5.20E+08	8.71600334		
Silver Sulfadizine 1%	37	1.00E+05	0.03	1.23E+08	2.47E+08	8.39211047	6.97740632	1.120442676
	18	1.00E+05	0.03	6.00E+07	1.20E+08	8.07918125		
	12	1.00E+03	0.03	4.00E+05	8.00E+05	5.90308999		
	40	1.00E+03	0.03	1.33E+06	2.67E+06	6.42596873		
	32	1.00E+02	0.03	1.07E+05	2.13E+05	5.32905872		
	18	1.00E+05	0.03	6.00E+07	1.20E+08	8.07918125		
	54	1.00E+03	0.03	1.80E+06	3.60E+06	6.55630250		
	17	1.00E+04	0.03	5.67E+06	1.13E+07	7.05435766		
Neosporin	27	1.00E+05	0.03	9.00E+07	1.80E+08	8.25527251	7.65111492	0.326611507
	25	1.00E+04	0.03	8.33E+06	1.67E+07	7.22184875		
	52	1.00E+04	0.03	1.73E+07	3.47E+07	7.53991208		
	43	1.00E+04	0.03	1.43E+07	2.87E+07	7.45737720		
	106	1.00E+04	0.03	3.53E+07	7.07E+07	7.84921461		
	11	1.00E+05	0.03	3.67E+07	7.33E+07	7.86530143		
	38	1.00E+04	0.03	1.27E+07	2.53E+07	7.40369234		
	62	1.00E+04	0.03	2.07E+07	4.13E+07	7.61630043		
Gentamicin 1%	0	1.00E+00	0.03	0.00E+00	0.00E+00	0	0.00000000	0
	0	1.00E+00	0.03	0.00E+00	0.00E+00	0		
	0	1.00E+00	0.03	0.00E+00	0.00E+00	0		
	0	1.00E+00	0.03	0.00E+00	0.00E+00	0		
	0	1.00E+00	0.03	0.00E+00	0.00E+00	0		
	0	1.00E+00	0.03	0.00E+00	0.00E+00	0		
	0	1.00E+00	0.03	0.00E+00	0.00E+00	0		
	0	1.00E+00	0.03	0.00E+00	0.00E+00	0		
CZ-01179 0.5%	0	1.00E+00	0.03	0.00E+00	0.00E+00	0	0.00000000	0
	0	1.00E+00	0.03	0.00E+00	0.00E+00	0		
	0	1.00E+00	0.03	0.00E+00	0.00E+00	0		
	0	1.00E+00	0.03	0.00E+00	0.00E+00	0		
	0	1.00E+00	0.03	0.00E+00	0.00E+00	0		
	0	1.00E+00	0.03	0.00E+00	0.00E+00	0		
	0	1.00E+00	0.03	0.00E+00	0.00E+00	0		

	0	1.00E+00	0.03	0.00E+00	0.00E+00	0		
CZ-01179 1%	0	1.00E+00	0.03	0.00E+00	0.00E+00	0	0.00000000	0
	0	1.00E+00	0.03	0.00E+00	0.00E+00	0		
	0	1.00E+00	0.03	0.00E+00	0.00E+00	0		
	0	1.00E+00	0.03	0.00E+00	0.00E+00	0		
	0	1.00E+00	0.03	0.00E+00	0.00E+00	0		
	0	1.00E+00	0.03	0.00E+00	0.00E+00	0		
	0	1.00E+00	0.03	0.00E+00	0.00E+00	0		
	0	1.00E+00	0.03	0.00E+00	0.00E+00	0		
CZ-01179 2%	0	1.00E+00	0.03	0.00E+00	0.00E+00	0	0.00000000	0
	0	1.00E+00	0.03	0.00E+00	0.00E+00	0		
	0	1.00E+00	0.03	0.00E+00	0.00E+00	0		
	0	1.00E+00	0.03	0.00E+00	0.00E+00	0		
	0	1.00E+00	0.03	0.00E+00	0.00E+00	0		
	0	1.00E+00	0.03	0.00E+00	0.00E+00	0		
	0	1.00E+00	0.03	0.00E+00	0.00E+00	0		
	0	1.00E+00	0.03	0.00E+00	0.00E+00	0		
