# Supplementary material for: Examination of a first-in-class bis-dialkylnorspermidine-terphenyl antibiotic in topical formulation against mono and polymicrobial biofilms
Source: PLoS One. 2020 Oct 19;15(10):e0234832. doi: 10.1371/journal.pone.0234832 (PMC7571676; doi:10.1371/journal.pone.0234832)
Supplement: S3 Table — (PDF) [file pone.0234832.s003.pdf]

Supplemental Table 3: Raw microbiological data for polymicrobial biofilms composed of MRSA and *Pseudomonas aeruginosa* (ATCC 27853)

Treatment	Incoulate	CFU	Dilution Factor 1 (scientific notation)	Dilution Factor 2 (1:20 for final pipet step)	CFU/mL	CFU/sample	Log_{10} Transformed	Average	Standard Deviation
**Collagen (+ controls)**	**MRSA**	43	1.00E+06	0.03	1.43E+09	2.87E+09	9.45737720	9.05987769	0.502401318
		64	1.00E+06	0.03	2.13E+09	4.27E+09	9.63008871		
		65	1.00E+06	0.03	2.17E+09	4.33E+09	9.63682210		
		56	1.00E+06	0.03	1.87E+09	3.73E+09	9.57209677		
		72	1.00E+06	0.03	2.40E+09	4.80E+09	9.68124124		
		51	1.00E+05	0.03	1.70E+08	3.40E+08	8.53147892		
		44	1.00E+05	0.03	1.47E+08	2.93E+08	8.46736142		
		43	1.00E+05	0.03	1.43E+08	2.87E+08	8.45737720		
		88	1.00E+05	0.03	2.93E+08	5.87E+08	8.76839141		
		18	1.00E+06	0.03	6.00E+08	1.20E+09	9.07918125		
		76	1.00E+05	0.03	2.53E+08	5.07E+08	8.70472233		
		81	1.00E+05	0.03	2.70E+08	5.40E+08	8.73239376		
	P. aeruginosa	42	1.00E+04	0.03	1.40E+07	2.80E+07	7.44715803	7.54858641	0.490463837
		15	1.00E+05	0.03	5.00E+07	1.00E+08	8.00000000		
		43	1.00E+04	0.03	1.43E+07	2.87E+07	7.45737720		
		63	1.00E+04	0.03	2.10E+07	4.20E+07	7.62324929		
		40	1.00E+04	0.03	1.33E+07	2.67E+07	7.42596873		
		33	1.00E+04	0.03	1.10E+07	2.20E+07	7.34242268		
		25	1.00E+04	0.03	8.33E+06	1.67E+07	7.22184875		
		9	1.00E+04	0.03	3.00E+06	6.00E+06	6.77815125		
		5	1.00E+05	0.03	1.67E+07	3.33E+07	7.52287875		
		10	1.00E+06	0.03	3.33E+08	6.67E+08	8.82390874		
		35	1.00E+04	0.03	1.17E+07	2.33E+07	7.36797679		
		56	1.00E+04	0.03	1.87E+07	3.73E+07	7.57209677		
**Silver Sulfadizine 1%**	**MRSA**	36	1.00E+01	0.03	1.20E+04	2.40E+04	4.38021124	4.86050348	0.529250637
		17	1.00E+01	0.03	5.67E+03	1.13E+04	4.05435766		
		77	1.00E+01	0.03	2.57E+04	5.13E+04	4.71039947		
		36	1.00E+02	0.03	1.20E+05	2.40E+05	5.38021124		
		21	1.00E+02	0.03	7.00E+04	1.40E+05	5.14612804		
		60	1.00E+02	0.03	2.00E+05	4.00E+05	5.60205999		
		18	1.00E+02	0.03	6.00E+04	1.20E+05	5.07918125		
		51	1.00E+01	0.03	1.70E+04	3.40E+04	4.53147892		
		83	1.00E+01	0.03	2.77E+04	5.53E+04	4.74298683		
		0	1.00E+01	0.03	0.00E+00	0.00E+00	0.00000000		

	P. aeruginosa	63	1.00E+01	0.03	2.10E+04	4.20E+04	4.62324929	3.80886035	1.602136958
		17	1.00E+02	0.03	5.67E+04	1.13E+05	5.05435766		
		29	1.00E+01	0.03	9.67E+03	1.93E+04	4.28630674		
		70	1.00E+00	0.03	2.33E+03	4.67E+03	3.66900678		
		20	1.00E+01	0.03	6.67E+03	1.33E+04	4.12493874		
		14	1.00E+01	0.03	4.67E+03	9.33E+03	3.97003678		
**Neosporin**	**MRSA**	2	1.00E+05	0.05	4.00E+06	8.00E+06	6.90308999	6.63674579	0.320216018
		26	1.00E+04	0.05	5.20E+06	1.04E+07	7.01703334		
		8	1.00E+04	0.05	1.60E+06	3.20E+06	6.50514998		
		6	1.00E+04	0.05	1.20E+06	2.40E+06	6.38021124		
		11	1.00E+04	0.05	2.20E+06	4.40E+06	6.64345268		
		23	1.00E+04	0.05	4.60E+06	9.20E+06	6.96378783		
		10	1.00E+04	0.05	2.00E+06	4.00E+06	6.60205999		
		3	1.00E+04	0.05	6.00E+05	1.20E+06	6.07918125		
	P. aeruginosa	96	1.00E+05	0.05	1.92E+08	3.84E+08	8.58433122	8.11840363	0.411814366
		51	1.00E+05	0.05	1.02E+08	2.04E+08	8.30963017		
		19	1.00E+05	0.05	3.80E+07	7.60E+07	7.88081359		
		15	1.00E+05	0.05	3.00E+07	6.00E+07	7.77815125		
		33	1.00E+05	0.05	6.60E+07	1.32E+08	8.12057393		
		73	1.00E+05	0.05	1.46E+08	2.92E+08	8.46538285		
		6	1.00E+05	0.05	1.20E+07	2.40E+07	7.38021124		
		67	1.00E+05	0.05	1.34E+08	2.68E+08	8.42813479		
**Gentamicin 1%**	**MRSA**	14	1.00E+01	0.03	4.67E+03	9.33E+03	3.97003678	3.84676911	0.312803775
		53	1.00E+00	0.03	1.77E+03	3.53E+03	3.54818461		
		52	1.00E+00	0.03	1.73E+03	3.47E+03	3.53991208		
		61	1.00E+00	0.03	2.03E+03	4.07E+03	3.60923858		
		68	1.00E+00	0.03	2.27E+03	4.53E+03	3.65641765		
		14	1.00E+01	0.03	4.67E+03	9.33E+03	3.97003678		
		17	1.00E+01	0.03	5.67E+03	1.13E+04	4.05435766		
		40	1.00E+01	0.03	1.33E+04	2.67E+04	4.42596873		
	P. aeruginosa	0	1.00E+00	0.03	0.00E+00	0.00E+00	0	0.00000000	0
		0	1.00E+00	0.03	0.00E+00	0.00E+00	0		
		0	1.00E+00	0.03	0.00E+00	0.00E+00	0		
		0	1.00E+00	0.03	0.00E+00	0.00E+00	0		
		0	1.00E+00	0.03	0.00E+00	0.00E+00	0		
		0	1.00E+00	0.03	0.00E+00	0.00E+00	0		
		0	1.00E+00	0.03	0.00E+00	0.00E+00	0		
		0	1.00E+00	0.03	0.00E+00	0.00E+00	0		
		0	1.00E+00	0.03	0.00E+00	0.00E+00	0		
		0	1.00E+00	0.03	0.00E+00	0.00E+00	0		

**CZ-01179 0.5%**	**MRSA**	0	1.00E+00	0.03	0.00E+00	0.00E+00	0	0.00000000	0
		0	1.00E+00	0.03	0.00E+00	0.00E+00	0		
		0	1.00E+00	0.03	0.00E+00	0.00E+00	0		
		0	1.00E+00	0.03	0.00E+00	0.00E+00	0		
		0	1.00E+00	0.03	0.00E+00	0.00E+00	0		
		0	1.00E+00	0.03	0.00E+00	0.00E+00	0		
	P. aeruginosa	0	1.00E+00	0.03	0.00E+00	0.00E+00	0	0.00000000	0
		0	1.00E+00	0.03	0.00E+00	0.00E+00	0		
		0	1.00E+00	0.03	0.00E+00	0.00E+00	0		
		0	1.00E+00	0.03	0.00E+00	0.00E+00	0		
		0	1.00E+00	0.03	0.00E+00	0.00E+00	0		
		0	1.00E+00	0.03	0.00E+00	0.00E+00	0		
		0	1.00E+00	0.03	0.00E+00	0.00E+00	0		
		0	1.00E+00	0.03	0.00E+00	0.00E+00	0		
**CZ-01179 1%**	**MRSA**	0	1.00E+00	0.03	0.00E+00	0.00E+00	0	0.00000000	0
		0	1.00E+00	0.03	0.00E+00	0.00E+00	0		
		0	1.00E+00	0.03	0.00E+00	0.00E+00	0		
		0	1.00E+00	0.03	0.00E+00	0.00E+00	0		
		0	1.00E+00	0.03	0.00E+00	0.00E+00	0		
		0	1.00E+00	0.03	0.00E+00	0.00E+00	0		
		0	1.00E+00	0.03	0.00E+00	0.00E+00	0		
		0	1.00E+00	0.03	0.00E+00	0.00E+00	0		
	P. aeruginosa	0	1.00E+00	0.03	0.00E+00	0.00E+00	0	0.00000000	0
		0	1.00E+00	0.03	0.00E+00	0.00E+00	0		
		0	1.00E+00	0.03	0.00E+00	0.00E+00	0		
		0	1.00E+00	0.03	0.00E+00	0.00E+00	0		
		0	1.00E+00	0.03	0.00E+00	0.00E+00	0		
		0	1.00E+00	0.03	0.00E+00	0.00E+00	0		
		0	1.00E+00	0.03	0.00E+00	0.00E+00	0		
		0	1.00E+00	0.03	0.00E+00	0.00E+00	0		
**CZ-01179 2%**	**MRSA**	0	1.00E+00	0.03	0.00E+00	0.00E+00	0	0.00000000	0
		0	1.00E+00	0.03	0.00E+00	0.00E+00	0		
		0	1.00E+00	0.03	0.00E+00	0.00E+00	0		
		0	1.00E+00	0.03	0.00E+00	0.00E+00	0		
		0	1.00E+00	0.03	0.00E+00	0.00E+00	0		
		0	1.00E+00	0.03	0.00E+00	0.00E+00	0		
		0	1.00E+00	0.03	0.00E+00	0.00E+00	0		
		0	1.00E+00	0.03	0.00E+00	0.00E+00	0		
		0	1.00E+00	0.03	0.00E+00	0.00E+00	0		
		0	1.00E+00	0.03	0.00E+00	0.00E+00	0		

P. aeruginosa	0	1.00E+00	0.03	0.00E+00	0.00E+00	0	0.00000000	0
	0	1.00E+00	0.03	0.00E+00	0.00E+00	0		
	0	1.00E+00	0.03	0.00E+00	0.00E+00	0		
	0	1.00E+00	0.03	0.00E+00	0.00E+00	0		
	0	1.00E+00	0.03	0.00E+00	0.00E+00	0		
	0	1.00E+00	0.03	0.00E+00	0.00E+00	0		
